# Poster Session II - A313 LOWER VISCERAL FAT ASSOCIATES WITH ACTIVE DISEASE IN MEN BUT NOT WOMEN WITH INFLAMMATORY BOWEL DISEASES

**DOI:** 10.1093/jcag/gwaf042.313

**Published:** 2026-02-13

**Authors:** B Maracle, D Hazra, K Novak, C Lu, G G Kaplan, M Raman, J Besney, R Reji, A AlDarwish, C Seow, R Ingram, C Ma, R Panaccione, J St-Pierre

**Affiliations:** Medicine, University of Calgary, Calgary, AB, Canada; University of Calgary, Calgary, AB, Canada; Medicine, University of Calgary, Calgary, AB, Canada; Medicine, University of Calgary, Calgary, AB, Canada; Medicine, University of Calgary, Calgary, AB, Canada; Medicine, University of Calgary, Calgary, AB, Canada; Medicine, University of Calgary, Calgary, AB, Canada; Division of Gastroenterology and Hepatology, University of Calgary, Calgary, AB, Canada; Medicine, University of Calgary, Calgary, AB, Canada; Medicine, University of Calgary, Calgary, AB, Canada; Medicine, University of Calgary, Calgary, AB, Canada; Medicine, University of Calgary, Calgary, AB, Canada; Medicine, University of Calgary, Calgary, AB, Canada; Medicine, University of Calgary, Calgary, AB, Canada

## Abstract

**Background:**

Visceral adipose tissue (VAT) is a metabolically active organ that modulates systemic and intestinal inflammation. While VAT expansion has been linked to metabolic dysfunction in inflammatory bowel disease (IBD), the influence of sex-specific adipose distribution remains largely unexplored.

**Aims:**

We aimed to characterize the relationship between VAT measured by ultrasound (US) and disease activity, stratified by sex.

**Methods:**

We conducted a cross-sectional, single-centre study of adults with IBD between January 2024 and August 2025. VAT thickness was measured by US. Clinical data was collected, along with clinical indices, biochemical markers and endoscopic findings. Composite disease activity was defined as active inflammation on ≥ 1 of the following within 3 months: IUS (bowel wall thickness >3 mm and/or hyperemia), endoscopic activity, CRP >8mg/L, fecal calprotectin >250mcg/g, or symptomatic activity. Continuous variables were analyzed using one-way ANOVA with Tukey’s post-hoc test for multiple comparisons when overall significance was achieved, and categorical variables were analyzed using the Freeman-Halton test. Analyses were stratified by sex to evaluate sex-specific associations between VAT and disease activity.

**Results:**

124 patients with IBD were included (59% Crohn’s disease; 60% male; median age 43 years [IQR 32–61]). Median BMI was 25.2 kg/m2 (IQR 22.4–29.1), with similar distribution between sexes (men 25.8 [23.1–29.4]; women 24.3 [21.5–28.3]). Median VAT thickness was higher in men (33.7mm [IQR 23.4-53.0mm]) than in women (28.6mm [IQR 13.7-49.4mm]). VAT correlated strongly with BMI in both sexes (men: Rho=0.66, p < 0.0001; women: Rho=0.58, p < 0.0001). Among men, VAT thickness was significantly lower in those with active disease compared to those in remission (median 27.5mm vs 44.0mm; p = 0.0008 at 1 month; p = 0.0002 at 3 months). In women, VAT did not differ significantly between active and inactive disease (median 29.1mm vs 27.8mm; p = 0.67 at 1 month; p = 0.74 at 3 months). VAT was not associated with age at diagnosis, disease duration, HbA1c, lipid profile, metabolic disease, or corticosteroid exposure in either sex.

**Conclusions:**

VAT thickness measured by US correlates with BMI in both men and women but shows a sex-specific relationship with inflammatory activity. These findings suggest that VAT may reflect a disease-modifying metabolic phenotype in men and highlight the need for sex-specific reference ranges and prospective validation of VAT as a biomarker.

A313 Table 1: VAT thickness by disease activity and sex in patients with IBD

**Funding Agencies:**

Dr Keith MacCannell Rising Researcher Scholar Award Endowment

**Table:** 

	Active Disease median VAT, mm [IQR]	Inactive Disease median VAT, mm [IQR]	p-value
Men	27.5 [19.2-35.8]	44.0 [31.0-59.0]	0.0008
Women	29.1 [17.3-41.4]	27.8 [16.0-39.6]	0.67

